# The association between stress, emotional states, and tinnitus: a mini-review

**DOI:** 10.3389/fnagi.2023.1131979

**Published:** 2023-05-03

**Authors:** Jayaditya Devpal Patil, Manar Abdulkarim Alrashid, Ayah Eltabbakh, Salim Fredericks

**Affiliations:** ^1^Department of Surgery, University Hospitals of Leicester NHS Trust, Leicester, United Kingdom; ^2^Royal College of Surgeons in Ireland, Al Muharraq, Bahrain

**Keywords:** stress, tinnitus, HPA axis, emotional states, anxiety, depression, PTSD, chronic stress

## Abstract

Extensive literature supporting the view of tinnitus induced stress in patients is available. However, limited evidence has been produced studying the opposite, that is, does stress cause tinnitus? The hypothalamus pituitary adrenal axis, one of the main neuroendocrine systems involved in stress response, is commonly disturbed in tinnitus patients. Patients with chronic tinnitus have been shown to develop abnormal responses to psycho-social stress, where the hypothalamus pituitary adrenal axis response is weaker and delayed, suggesting chronic stress contributes to the development of chronic tinnitus. The sympathetic branch of the autonomic nervous system also plays a major role in stress response and its chronic hyperactivity seems to be involved in developing tinnitus. Psycho-social stress has been shown to share the same probability of developing tinnitus as occupational noise and contributes to worsening tinnitus. Additionally, exposure to high stress levels and occupational noise doubles the likelihood of developing tinnitus. Interestingly, short-term stress has been shown to protect the cochlea in animals, but chronic stress exposure has negative consequences. Emotional stress also worsens pre-existing tinnitus and is identified as an important indicator of tinnitus severity. Although there is limited body of literature, stress does seem to play a vital role in the development of tinnitus. This review aims to highlight the association between stress, emotional states, and the development of tinnitus while also addressing the neural and hormonal pathways involved.

## Introduction

Tinnitus is a symptom characterized by a phantom auditory perception in the absence of an external stimulus (Henry et al., 2005). It is a common condition, with prevalence ranging from 8 to 25.3% in the United States of America. Population-based studies in other countries have reported similar prevalences, ranging from 4.6 to 30% (Khedr et al., 2010; Jalessi et al., 2013; Park and Moon, 2014). Chronic tinnitus, defined as the presence of tinnitus for more than 3 months, is more prevalent among seniors (12% after age 60) than in young adults (5% in the 20–30 age group) but can occur at any age (Chronic Tinnitus, 2006). In 1–3% of the general population, tinnitus is loud enough to affect the quality of life, causing sleep disturbance, work impairment, and psychiatric distress (Dobie, 2003). There are also concerns about future increases in the prevalence of tinnitus, due to increased exposure to loud music and leisure noise (Pienkowski, 2021).

The etiology of tinnitus has been extensively researched and common causes include noise induced hearing loss, presbycusis, Meniere’s disease, infectious causes, and neurological etiologies such as whiplash injury and acoustic neuroma. Tinnitus may also present as a side effect of medications such as salicylates, non-steroidal anti-inflammatory drugs, aminoglycoside antibiotics, loop diuretics, and chemotherapy agents (Han et al., 2009). Pathologic lesions in the auditory pathway or reduction in auditory nerve function can also cause tinnitus (Nuttall et al., 2004). Epigenetic processes, which involve phenotypic changes caused by modification of genetic expression, have recently been proposed as mechanisms behind hearing- loss-related syndromes, contributing to the pathogenesis of tinnitus (Mittal et al., 2020). Interestingly however, approximately 40% of patients cannot identify any underlying cause for their tinnitus (Henry, 2004).

Patients suffering from tinnitus perceive their symptoms as stressful and are also often impaired by psychological problems like depression, anxiety, difficulty concentrating, and insomnia (Zöger et al., 2006; Milerová et al., 2013; Mohamad et al., 2016; Tegg-Quinn et al., 2016). About 10–60% of chronic tinnitus patients suffer from depressive disorders and 28–45% present with clinically relevant symptoms of anxiety (Andersson, 2002; Reynolds et al., 2004). It has also been observed that many tinnitus patients present with psychological or psychiatric distress before or during the onset and evolution of tinnitus, suggesting a relationship (Ciminelli et al., 2018).

Although tinnitus itself is known to cause discomfort and stress to the patient, research studying the role of stress and emotional states in the development of tinnitus and their effect on the clinical course of pre-existing tinnitus has been limited and somewhat mixed. This mini-review highlights available literature assessing the potential of stress and emotional states to cause tinnitus while also discussing their impact on pre-existing tinnitus and shedding light on associated neural and hormonal mechanisms of action. Results from this review will allow researchers and readers alike to better understand the association between stress, emotional states, and tinnitus and serve as a template for potential interventions to prevent onset of tinnitus secondary to stress and improve prognosis.

## Stress and tinnitus

**Figure 1:**
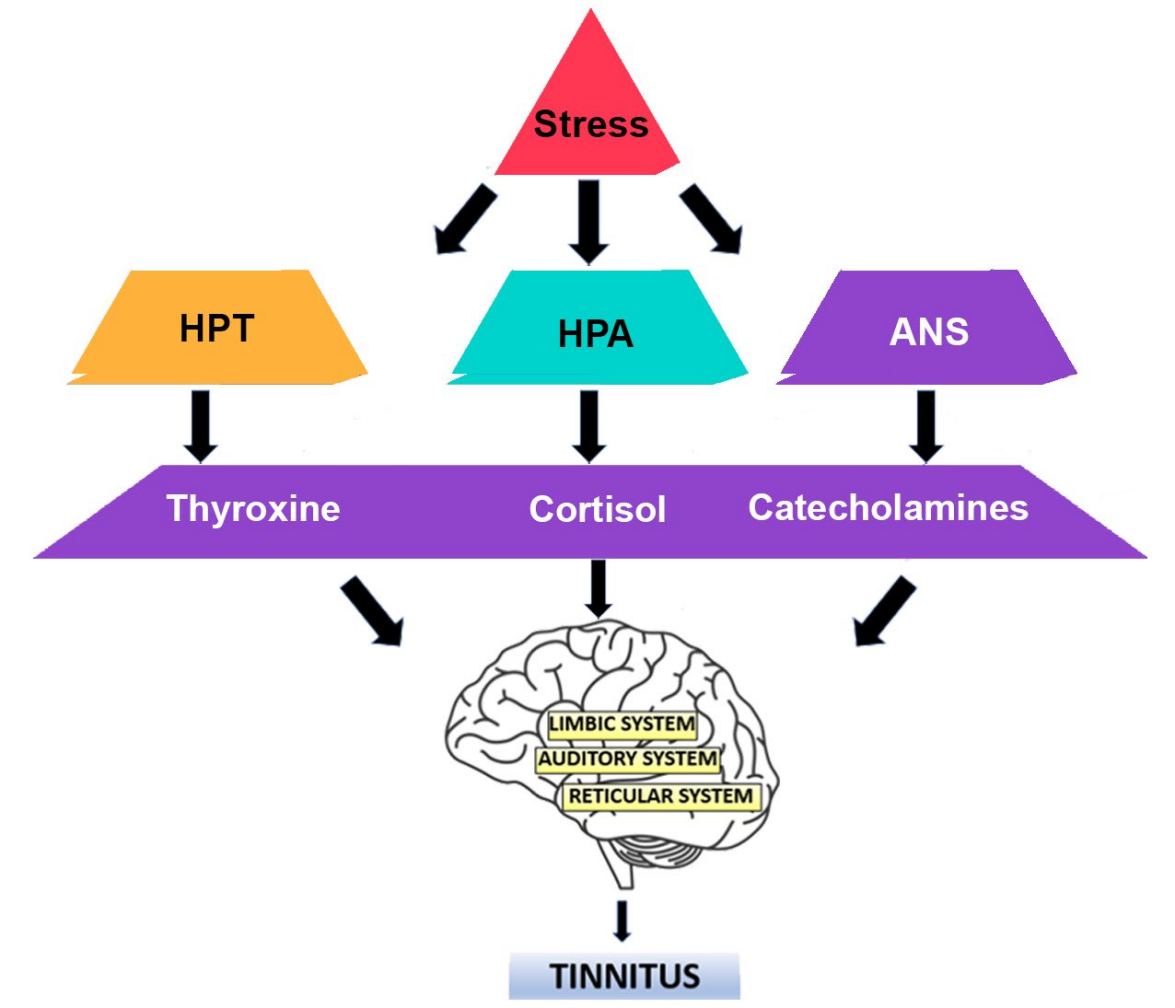
The response to stress involves various neural and hormonal pathways. The hypothalamus-pituitary-thyroid (HPT) axis, hypothalamus-pituitary–adrenal (HPA) axis, and the autonomic nervous system (ANS) have been implicated in the development of tinnitus.

The response to stress involves various neural and hormonal pathways, namely, the hypothalamus-pituitary-thyroid (HPT) axis (Mebis and van den Berghe, 2009), hypothalamus-pituitary–adrenal (HPA) axis (Lupien et al., 2007), and the autonomic nervous system (ANS) (Ulrich-Lai and Herman, 2009). These relationships are summarized in Figure 1. The role of these pathways in response to stress has been researched and their interaction with the pathogenesis of tinnitus has also been reported. The nervous system adapts to stress by neuronal plasticity, which plays a key role in the development of tinnitus (Eggermont and Roberts, 2004; Eggermont, 2015). This has also been researched in animals, where stress alters synaptic plasticity in the hippocampus in rats (Yeh et al., 2012). Reduced stimuli input is an important promoter of neuronal plasticity expression and tinnitus is commonly seen with hearing loss or auditory nerve injury, thus suggesting a link between neuronal plasticity and tinnitus onset (Bauer et al., 2007; Møller, 2007). The limbic system has been linked with tinnitus where the hippocampus, amygdala, and prefrontal cortex have been shown to potentially be affected by cortisol release following response to stress (Eggermont and Roberts, 2004; Simoens and Hébert, 2012; Eggermont, 2015). Chronic activation of the HPA-axis and subsequent elevations in plasma cortisol has been associated with several conditions, including anxiety and depression (Lupien et al., 2007). As an alternative to measuring cortisol plasma concentrations, a recent study has suggested hair content of cortisol may be a better biometric measure of the stress response (Basso et al., 2022). Basso et al. (2022) compared a panel of psychometric measures of tinnitus and stress with the two biomarkers of stress: cortisol and brain-derived neurotrophic factor (BDNF), using hair samples from patients with tinnitus. They reported higher perceived tinnitus loudness to be associated with higher hair-cortisol and lower hair-BDNF. Further, higher tinnitus-related distress was associated with lower hair-BDNF. However, a longitudinal study from the same research group found no association between tinnitus-related distress and perceived stress with either of these biomarkers (Basso et al., 2022). The authors conclude that further studies are needed to investigate hair-biomarkers with tinnitus patients (Basso et al., 2022).

Stress also affects the auditory system. One animal study measuring neural activity in response to stress in the auditory cortex of rats reported a direct link between the two, wherein sound-evoking activity in the auditory cortex was enhanced in response to stress (Ma et al., 2015). These findings, however, will need to be reproduced in human trials to conclude the same.

Stress factors and hormones in the endocrine system can affect the limbic, reticular, and auditory systems and interactions within these systems have been proposed to induce tinnitus and/or hyperacusis (Al-Mana et al., 2008; Kraus and Canlon, 2012). Patients with tinnitus have been reported to have elevated hormones such as norepinephrine and the serotonin metabolite 5-hydroxy indole acetic acid (5- HIAA) (Kim et al., 2014). Immunological dysregulation has also been associated with the two (Hébert et al., 2004; Hébert and Lupien, 2007; Lupien et al., 2009). Both stress responses and tinnitus share interactions with the HPA axis and the ANS, which has been extensively reported by Mazurek et al. (2019). A positive correlation of the sympathetic nervous system has been reported in tinnitus related distress (Datzov et al., 1999) while parasympathetic tone was found to be increased in cases of tinnitus suppression (Matsushima et al., 1996). Other studies, however, have failed to show significant findings (Değirmenci et al., 2014).

The HPA axis is altered in tinnitus patients, where response to stress is delayed and sub-optimal (Mazurek et al., 2012). Szczepek and Mazurek (2021) reviewed stress-induced mechanisms affecting cochlear physiology in the context of tinnitus generation and reported HPA axis- induced actions on mineralocorticoid and glucocorticoid receptors and altered gene expression in the cochlea. The corticosteroids released upon HPA activation may contribute to the N-methyl-D-aspartate (NMDA)/aminomethylphosphonic acid (AMPA) imbalance, further supporting the role of the HPA axis in the onset of stress-induced tinnitus. The sympathetic-adreno-medullar (SAM) axis was also proposed to increase blood pressure, inducing degenerative microvascular changes in the cochlea likely leading to hypoxia and potentially damaging auditory hair cells and spiral ganglion neurons. The authors, however, have advised further investigations to confirm this hypothesis. Gamma-aminobutyric acid (GABA)-related genetic changes may also be responsible for decreased auditory function and HPA inhibition as stress on the HPA axis can be hampered by GABA (Mazurek et al., 2015). These findings display the complex pathways involved in stress-induced tinnitus.

Some literature involving animal and human trials has highlighted an association between stress and the onset of tinnitus. One animal study assessing low-frequency hearing loss in prenatally stressed rats concluded prenatal stress to cause low-frequency hearing loss (Kadner et al., 2006). Prenatal stress has also been associated with dysregulation of the HPA axis (Hougaard et al., 2005; Koenig et al., 2005). The study in question reported acute short-term stress to protect the cochlea in animal models, however, this has not been replicated in humans (Wang and Liberman, 2002; Tahera et al., 2007). Another group studying changes in behavior and brain glucose metabolism in rats in a chronic mild stress model of depression with PET imaging reported activation of the left auditory cortex and deactivation of the left inferior colliculus in stressed animals after 4 weeks (Hu et al., 2010). Though these findings do not directly correlate with tinnitus, the association with hearing loss and interaction with the auditory cortex, respectively, suggests an underlying mechanism with tinnitus onset. An experimental study evaluating whether tinnitus can develop due to, or be aggravated by stress, in rats, found reduced gap prepulse inhibition of the acoustic startle (GPIAS) reflex, a reliable indicator of tinnitus development in animals (Kim et al., 2021). The study also reported decreased immunofluorescence expression of GABA A receptor α1 and increased NMDA receptor 1 immunofluorescence expression in the hippocampus in the group exposed to both noise and stress. This suggests an imbalance in excitatory and inhibitory neurotransmitters in the hippocampus to be the mechanism responsible (Kim et al., 2021). The inferior colliculus is also affected by stress where atrophy of the inferior colliculus in rat brains was reported by a study assessing the effect of chronic immobilization stress isolation in the auditory and visual regions (Dagnino-Subiabre et al., 2005). Similarly, another study looking at the effect of stress on the auditory system in Wistar rats concluded significant temporary reductions in evoked auditory potentials and increase in expression of inflammatory genes in the inferior colliculus (Mazurek et al., 2010, 2012). These findings support the potentially detrimental effects of stress on the auditory system. Although no direct correlation with tinnitus was evaluated, given the results, development of tinnitus seems very plausible and further research in both animals and humans may support this.

Long-term stress exposure has also been suggested as a key predisposing factor for tinnitus (Simoens and Hébert, 2012). A study conducted in Germany evaluating the extent of chronic stress as an influencing factor among tinnitus sufferers concluded that about 25% of tinnitus sufferers considered chronic stress the main reason for their tinnitus (Schaaf et al., 2014). The data concluded comprise patient reported questionnaires and do not directly associate stress duration with tinnitus; however, the high response rate accounting for chronic stress supports a potential relationship. One study assessing the prevalence of hearing complaints and tinnitus regarding different work-and health related stressors found a nearly linear correlation between tinnitus and the duration of stress (Hasson et al., 2011). This study, however, only described prevalence without directly studying tinnitus and stress duration. Additionally, the study considered multiple stressors, not allowing researchers to conclude a single implicating factor. A survey evaluating the influence of noise and stress on the probability of tinnitus in the general population reported stress to be almost as important as occupational noise exposure regarding discomfort level secondary to tinnitus. Although the study included *n* = 12,166 responders, patient hearing loss was not taken into account and the survey could not prove a direct association (Baigi et al., 2011). One cross-sectional study investigated 658 users of the “TrackYourTinnitus” smartphone application and reported a direct effect of stress level on tinnitus loudness and tinnitus distress, where stress levels acted as partial mediators (Probst et al., 2016). Hébert et al. (2004) demonstrated that on completing social stress tasks, tinnitus patients with high stress levels had higher serum cortisol levels and subjective feelings of stress and tinnitus severity. In patients with chronic tinnitus, exposure to an acute stressor induced sustained cortisol levels or a reduced cortisol response and subjective experiences of higher stress. This suggests that chronic stress may contribute to the development of chronic tinnitus (Hébert and Lupien, 2007). Taken together, these studies suggest the duration of stress plays a role in the status of tinnitus severity but the data analyzed predominantly consisted of self-ratings and did not directly assess a quantitative association. Randomized controlled trials and other research may be useful in confirming this relationship.

Stress may also affect the status of pre-existing tinnitus and potentially alter overall clinical course. A study evaluating depression, anxiety, and stress associated with tinnitus patients reported a direct correlation between stress severity, and the severity and duration of tinnitus (Baigi et al., 2011). A review on updated literature on emotional stress influence on the functioning and homeostasis of the auditory system highlighted unpublished data (S Herbert) which showed more than 53.6% of tinnitus patients reported recurrence of symptoms during stressful periods and 52.8% reported worsening of symptoms during these periods (Mazurek et al., 2012, 2015). The results, however, do prove a causal relationship and require further research. Stress has also been suggested as a vital risk factor in the transition from mild to severe tinnitus (Gomaa et al., 2014). A study evaluating the effect of stressful life events as precipitating or exacerbating factors for tinnitus sensation found the tinnitus handicap inventory scores (a self-reported measure to determine perceived tinnitus handicap severity) of the study group to be significantly higher when compared to control, suggesting stress to worsen tinnitus (Yıldırım et al., 2017).

A similar cross-sectional study evaluating the associations between hearing status and health regarding tinnitus reported emotional exhaustion as a predictor of tinnitus (Hébert et al., 2012; Brüggemann et al., 2016). Brüggemann et al. (2016) aimed to associate the grade of tinnitus-related distress with the psychological distress, physical, or psychological discomfort, and concluded hearing loss, perceived stress-related tension, pessimism, and concentration to be predictors of tinnitus-related distress. This could mean that physical and psychological stress can determine the level of distress secondary to tinnitus.

Although the pathogenesis is unclear, one study assessing the role of endogenous dynorphins and glutamate and NMDA receptors in stress-mediated Type-I auditory neural exacerbation of tinnitus concluded stress-activated release of dynorphins into the cochlea to potentiate the effects of glutamate, leading to hyperacusis, with acute exacerbation of chronic aberrant Type-I neural activity and worsening of the central auditory neural plasticity responsible for tinnitus perception (Sahley et al., 2013) However, further research will be required to identify the underlying pathophysiology of stress-induced worsening of tinnitus.

## Emotional states and tinnitus

**Figure 2:**
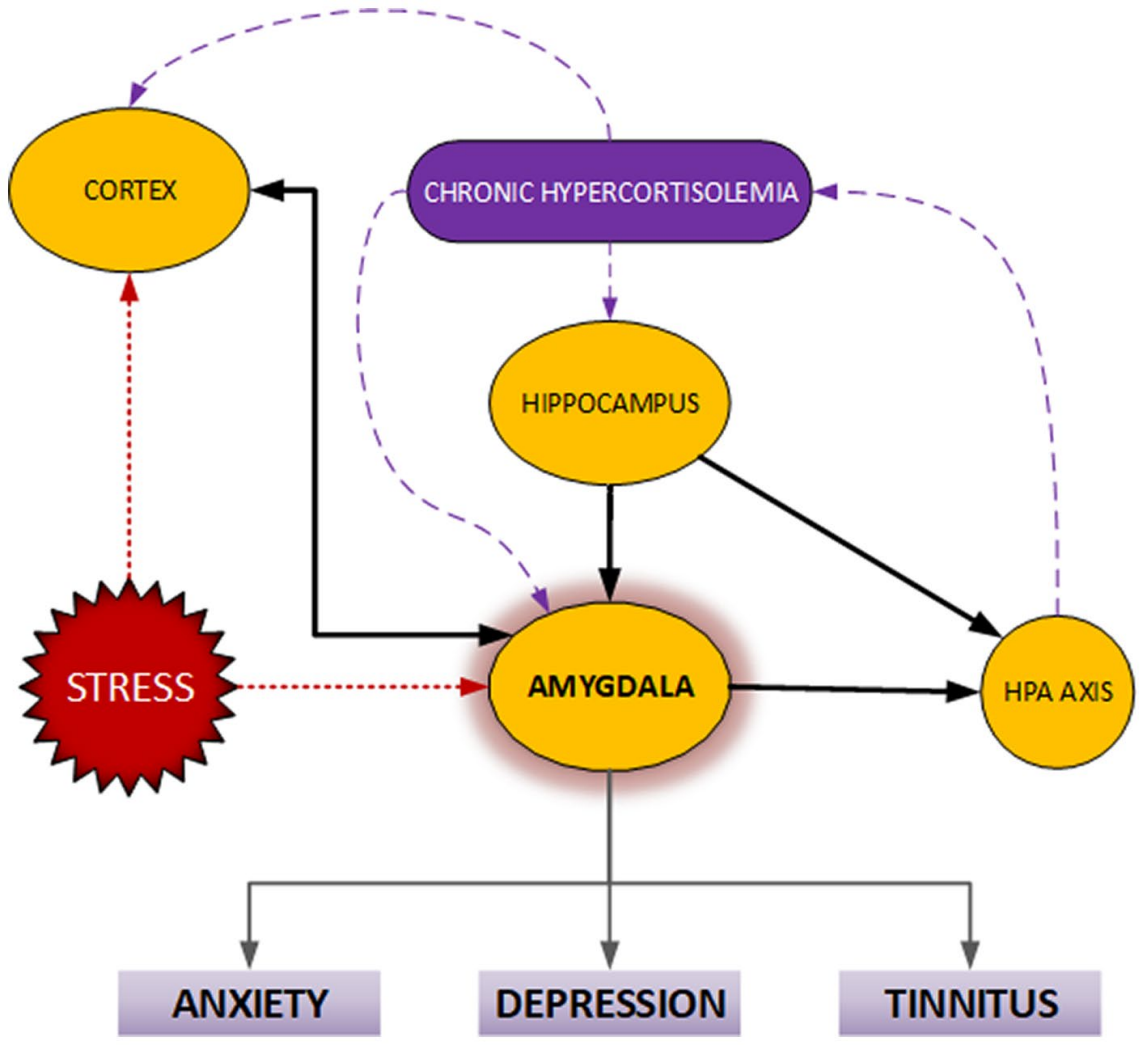
Disturbances of the hypothalamic–pituitary–adrenal axis (HPA-axis) are common to anxiety, depression, and tinnitus. The resulting chronically increased cortisol levels may influence limbic system components, particularly the amygdala. These areas are implicated in the anxiety response, symptoms of depression, and tinnitus.

Tinnitus has been associated with anxiety, depression, insomnia, and post-traumatic stress disorder (PTSD). These associations and pathways involved have been briefly discussed in Figure 2. High tinnitus severity has been linked to diagnosis with psychological conditions and positive correlation between tinnitus severity and increase in variables of anxiety, insomnia, and depression have also been reported (Beukes et al., 2021). Mood disorders have been linked to the dysfunction of neurotransmitters involved in the habituation process, which helps decrease tinnitus intrusiveness with time. A key role of the habituation process is to prevent overstimulation from harming the auditory system. This is achieved *via* complex neuronal circuits and multiple transmitter systems; acetylcholinergic, dopaminergic, GABA-ergic, nitric oxide, and serotonergic systems. The serotonergic system reacts to continuous stimulation by enforcing a “gain-control” between facilitating and inhibitory mechanisms. Thus, changes to neurotransmitters and dysfunction of habituation processes caused by various emotional states could explain the association with tinnitus (Al-Mana et al., 2008).

### Post-traumatic stress disorder and tinnitus

Both PTSD and tinnitus share decreased tolerance to loud noises, and tinnitus exacerbations in patients have been reported to occur due to reminiscence of triggering sounds from past trauma. Another association relates to the medication used in both disorders. Selective serotonin reuptake inhibitors (SSRIs) are the “front line” treatment options for PTSD and as both disorders share similar biochemical stimuli, the same medication may affect them in comparable ways. One study conducted on veterans to determine the correlation between tinnitus and PTSD reported that tinnitus loudness was exacerbated during periods of stress (Moring et al., 2018). Another study suggests that tinnitus may serve as a significant contributor to symptoms of PTSD, as they are caused by past traumatic events that included loud noises such as gunfire and explosions (Fagelson, 2007). Tinnitus and PTSD have also been shown to share alterations in neural anatomy where some reviews report physical changes to the thalamus, hippocampus, and amygdala, with reduced hippocampus vascularity and subsequent volume reduction being reported in patients of abuse and trauma (Shulman et al., 1995; Lockwood et al., 1998; Bremner, 2002; Cacace, 2003; Bremner, 2005). This association is further complicated by interaction between the amygdala and the medial pre-frontal cortex (Sotres-Bayon et al., 2004).

### Insomnia and tinnitus

A study conducted in an Audiology Department in the United Kingdom, reported nearly 70% of patients seeking help for tinnitus to have symptoms of insomnia. In addition, the emotional distress caused by tinnitus was more likely caused by the severity of insomnia (Aazh and Moore, 2019). Often, tinnitus therapies tend to improve insomnia complaints, since both conditions are associated with diseases such as depression and anxiety. Furthermore, some evidence suggests that both conditions induce the hyperactivation of the ANS, limbic system as well as the HPA axis (Wallhäusser-Franke et al., 2013). One animal study assessing brain activity, post-exposure to loud noises or systemic application of large dose salicylates, reported an increased number of immunoreactive neurons in the auditory cortex when compared to controls. In contrast, exposure to impulse noise led to prolonged *c-fos* expression, a marker of neuronal activity (Wallhäusser-Franke et al., 2013). Some studies have suggested psychological and physiological mechanisms to be similar in chronic tinnitus and primary insomnia, including dysfunctional beliefs, negative thoughts, and hyperarousal (Richter et al., 2021).

### Depression and tinnitus

Multiple studies have highlighted an association between depression and tinnitus. Some studies have suggested the anterior parietal area, the limbic system, comprising the anterior cingulate cortex, anterior insula, amygdala, and the hippocampal and para hippocampal area to all be potentially involved in the association between tinnitus and depressive mood (Lockwood et al., 1998; Besteher et al., 2019; Hu et al., 2021). Folmer et al. (1999) reported the current prevalence of depression in tinnitus patients at 27.8% and the lifetime prevalence at 34.6%. Another study looking at United States veterans revealed that 38% of those diagnosed with tinnitus were also diagnosed with depression (Martz et al., 2018). The mechanism of tinnitus caused by depression is thought to be the impairment of the habituation process. A study by Trevis et al. (2016) contradicts the relationship by studying 70 tinnitus patients and revealing their average Beck Depression Inventory (BDI) to be within a minimal range. However, the study did not use a control group for comparison. Assessing the results of another study that included a control group, a significant statistical difference between the BDI’s of the control and tinnitus groups is highlighted (Weidt et al., 2016). Although this demonstrates a difference between both groups, the BDI of the tinnitus group is still within the minimal range. However, only 42 tinnitus patients were used in this study compared to 70 in the former. Although there is evidence suggestive of an association between tinnitus and depression, further research with larger sample sizes and control groups will be required.

### Anxiety and tinnitus

Several studies have highlighted a link between tinnitus and anxiety. One study concluded that moderate or severe anxiety was experienced in 24% of tinnitus patients (Ciminelli et al., 2018). Another study reported that tinnitus patients who perceived their tinnitus as severe had an anxiety rate of 40.4% compared to the 10.6% reported by those who did not perceive their tinnitus as severe (Bhatt et al., 2017). This not only suggests an association between anxiety and tinnitus but also between anxiety and tinnitus severity. The mechanism behind this relationship still remains unclear. Kaltenbach, (2006) suggested an explanation by claiming that when the dorsal cochlear, which plays a significant role in producing norepinephrine and serotonin, is hyperactive, the locus coeruleus is stimulated leading to anxiety. Since injury of the cochlea often causes dorsal cochlear hyperactivity, such an explanation would clarify the relation between anxiety and tinnitus. However, there is no consensus between the studies that challenge this hypothesis. Karaaslan et al. (2020) concluded that despite tinnitus patients having higher anxiety scores compared to control groups, results are not statistically significant. On the contrary, another study reported statistical significance between cognitive concerns in both groups, with higher scores in tinnitus patients (Kumbul et al., 2022). One study conducted in Sweden suggesting the perception of tinnitus severity to be affected by anxiety disorders could explain discrepancies found in results (Holgers et al., 2005).

Various structures involved in tinnitus networks are also shared with anxiety disorders. The amygdala is commonly involved along with the insula and hippocampus (Cain and Ledoux, 2008; Craig, 2009; Kraus and Canlon, 2012; Ledoux, 2012). The locus coeruleus and the raphe nucleus are other structures also involved, predominantly mediating limbic system hyperresponsiveness, a phenomenon observed in both tinnitus and anxiety disorders (Pohl et al., 1987; Shulman et al., 1995; Lockwood et al., 1998; Tanaka et al., 2000; Etkin and Wager, 2007; Martin et al., 2009). These findings imply that the anxiety and tinnitus may share more in common than previously thought but further research is required to explore the relationship and pathophysiology of interaction between tinnitus and anxiety.

## Implications for treatment

A variety of interventions have been used to manage both tinnitus and stress caused by it. These range from psychological to psychopharmacological modalities with many studies supporting their effectiveness (Weber et al., 2002; Rief et al., 2005; Fornaro and Martino, 2010). Psychological interventions targeted toward stress management seem to play a vital role in tinnitus treatment, where one study suggests particular emphasis on early-stage intervention prior to chronic changes in neuronal plasticity (Ciminelli et al., 2018). Further studies both in animals and humans are strongly encouraged to establish conclusive evidence both for understanding pathophysiology and potential treatment outcomes. Studies focusing on short- and long-term outcomes will also be valuable in determining clinical progression of tinnitus, effects on preexisting tinnitus, and determining appropriate time frame for curative or preventive intervention.

## Conclusion

The nature of tinnitus is multifactorial and involves both auditory and emotional systems (Kaltenbach, 2011). The pathways involved in these systems inevitably have common threads and elements. Several associations between the two systems have been highlighted here. However, the current body of knowledge is not emphatic on causal links between mood states, stressors and tinnitus. A major consideration that must be considered when assessing the various studies associating tinnitus with stress and mood disorders is selection bias and collider bias. These have been acknowledged as sources of distorted associations between predictors and outcomes in this area (Couth et al., 2019; Lan et al., 2020). Also, correcting for confounding factors such as age and degree of hearing loss, the severity of depression, and anxiety has been challenging in existing studies. Future work is likely to be more cognizant of these issues.

Previously, there has been a focus in the research on psychometric rather than biometric reporting. This will likely change with improvements in laboratory assays and sampling methods such as hair sampling. Future studies may explore the relationship between tinnitus-related distress and biomarkers of mood states, and these should improve the understanding of both mood states and tinnitus.

Although major advances have been made in understanding the basic biology of tinnitus, this understanding has not translated well into the clinical setting. Future therapeutic approaches to tinnitus will be influenced by the elucidation of the neurochemistry and cellular plasticity involved in tinnitus pathophysiology (Guitton, 2012). In tandem with this evolving science, the mapping out pathways for stress, anxiety, and depression will likely guide future treatments for tinnitus. In order for the fundamental neuroscience of tinnitus to be translational, we predict that research will be performed in parallel or in conjunction with the fundamental neuroscience of stress and emotional states.

Collectively, evidence suggests stress plays a role in developing tinnitus and the worsening of pre-existing tinnitus. Further research combining auditory and emotional systems would be beneficial to establish this hypothesis and help guide treatment modalities, prevent tinnitus secondary to stress exposure, and predict the prognosis of patients with tinnitus.

## Author contributions

All authors listed have made a substantial, direct, and intellectual contribution to the work and approved it for publication.

## Conflict of interest

The authors declare that the research was conducted in the absence of any commercial or financial relationships that could be construed as a potential conflict of interest.

## Publisher’s note

All claims expressed in this article are solely those of the authors and do not necessarily represent those of their affiliated organizations, or those of the publisher, the editors and the reviewers. Any product that may be evaluated in this article, or claim that may be made by its manufacturer, is not guaranteed or endorsed by the publisher.
